# Supplementary material for: Photoprotective Agents Obtained from Aromatic Plants Grown in Colombia: Total Phenolic Content, Antioxidant Activity, and Assessment of Cytotoxic Potential in Cancer Cell Lines of Cymbopogon flexuosus L. and Tagetes lucida Cav. Essential Oils
Source: Plants (Basel). 2022 Jun 27;11(13):1693. doi: 10.3390/plants11131693 (PMC9269283; doi:10.3390/plants11131693)
Supplement: Supplementary file 1 [file plants-11-01693-s001.zip › plants-1743036-SM.pdf]

SUPPLEMENTARY INFORMATION

# Photoprotective agents obtained from aromatic plants grown in Colombia: total phenolic content, antioxidant activity, and assessment of cytotoxic potential in cancer cell lines of *Cymbopogon flexuosus* L. and *Tagetes lucida* Cav. essential oils

**Karina Caballero-Gallardo [1,2], Patricia Quintero-Rincón [1,2], Elena E. Stashenko [3] and Jesus Olivero-Verbel [1*]**

[1] Environmental and Computational Chemistry Group. School of Pharmaceutical Sciences, Zaragocilla Campus, University of Cartagena, Cartagena 130014, Colombia.
[2] Functional Toxicology Group. School of Pharmaceutical Sciences, Zaragocilla Campus, University of Cartagena, Cartagena 130014, Colombia.
[3] Center for Chromatography and Mass Spectrometry CROM-MASS, Research Center for Biomolecules CIBIMOL, School of Chemistry, Universidad Industrial de Santander, Bucaramanga 680006, Colombia.
* Correspondence: joliverov@unicartagena.edu.co (J.O.-V.) (Tel: 318-535-9815; Fax: 57-(5)-6699771)

**Contents**

I. **Supplementary Table S1.** Cytotoxic potential ($IC_{10}$ and $IC_{25}$) on HepG2 and Calu-1 cells.
II. **Supplementary Table S2.** EE(λ) x I(λ) constant values to wavelength determinate.
III. **Supplementary Table S3.** Erythema and pigmentation flux constant values on sunscreens to wavelength determinate.
IV. **Supplementary Table S4.** Classification of sunscreen according to erythema and pigmentation transmission indices.

I. **Supplementary Table S1.** Cytotoxic potential ($IC_{10}$ and $IC_{25}$) on HepG2 and Calu-1 cells.

| Essential oil | Cytotoxic potential derived of MTT viability assay (24 h) | | | | | |
|---|---|---|---|---|---|---|
| | HepG2 | | | Calu-1 | | |
| *Cymbopogon flexuosus* | **$IC_{10}$** | **$R^2$** | ***p*-value** | **$IC_{10}$** | **$R^2$** | ***p*-value** |
| | 29 | 0.986 | <0.0001 | 46 | 0.865 | 0.0072 |
| | **$IC_{25}$** | **$R^2$** | ***p*-value** | **$IC_{25}$** | **$R^2$** | ***p*-value** |
| | 46 | 0.986 | <0.0001 | 66 | 0.865 | 0.0072 |
| *Tagetes lucida* | **$IC_{10}$** | **$R^2$** | ***p*-value** | **$IC_{10}$** | **$R^2$** | ***p*-value** |
| | 105 | 0.912 | 0.0002 | 170 | 0.856 | 0.0010 |
| | **$IC_{25}$** | **$R^2$** | ***p*-value** | **$IC_{25}$** | **$R^2$** | ***p*-value** |
| | 167 | 0.912 | 0.0002 | 249 | 0.856 | 0.0010 |

IC. Inhibitory concentration (μg/mL).

**II. Supplementary Table S2.** EE(λ) x I(λ) constant values to wavelength determinate.

| Wavelength (λ, nm) | EE (λ)x I (λ) |
|---|---|
| 290 | 0.015 |
| 295 | 0.082 |
| 300 | 0.287 |
| 305 | 0.328 |
| 310 | 0.186 |
| 315 | 0.084 |
| 320 | 0.018 |

**III. Supplementary Table S3.** Erythema and pigmentation flux constant values on sunscreens to wavelength determinate.

| Wavelengh (λ; nm) | Flux of erythema (Fe) | Flux of pigmentation (Fp) |
|---|---|---|
| 290-295 | 11.390 | |
| 295-300 | 65.100 | |
| 300-305 | 100.000 | |
| 305-310 | 35.770 | |
| 310-315 | 0.973 | |
| 315-320 | 0.567 | |
| 320-325 | 337.500 | 10.790 |
| 325-330 | 0.289 | 10.200 |
| 330-335 | 0.129 | 0.936 |
| 335-340 | 0.046 | 0.798 |
| 340-345 | | 0.669 |
| 345-350 | | 0.570 |
| 350-355 | | 0.488 |
| 355-360 | | 0.456 |
| 360-365 | | 0.356 |
| 365-370 | | 0.310 |
| 370-375 | | 0.260 |
| Σ | 551.764 | 25.833 |

Constant values of Fe and Fp taken and modified from Sami et al. (2021).

**IV. Supplementary Table S4.** Classification of sunscreen according to erythema and pigmentation transmission indices.

| *Category* | UV transmission range (%) | |
|---|---|---|
| | *Erythema* | *Pigmentation* |
| Sunscreen | <1 | 3-40 |
| Extra protection | 1-6 | 42-86 |
| Standard tan | 6-12 | 45-86 |
| Quick tan | 10-18 | 45-86 |

**References**

Sami, F.J.; Soekamto, N.H.; Latip, J. Bioactivity profile of three of seaweed as an antioxidant, UV-protection as sunscreen and their correlation activity. Food Res. **2021**, 5(*1*): 441-447.
